# Effects on Engagement and Health Literacy Outcomes of Web-Based Materials Promoting Physical Activity in People With Diabetes: An International Randomized Trial

**DOI:** 10.2196/jmir.6601

**Published:** 2017-01-23

**Authors:** Ingrid Muller, Alison Rowsell, Beth Stuart, Victoria Hayter, Paul Little, Kristin Ganahl, Gabriele Müller, Gerardine Doyle, Peter Chang, Courtney R Lyles, Don Nutbeam, Lucy Yardley

**Affiliations:** ^1^ Department of Psychology University of Southampton Southampton United Kingdom; ^2^ University of Southampton Department of Health Sciences Southampton United Kingdom; ^3^ Primary Care and Population Sciences University of Southampton Southampton United Kingdom; ^4^ Gesundheit Österreich GmbH (Austrian Public Health Institute) Vienna Austria; ^5^ Centre for Evidence-based Healthcare University Hospital Carl Gustav Carus Technical University Dresden Dresden Germany; ^6^ UCD College of Business University College Dublin Dublin Ireland; ^7^ Taipei Medical University and National Taipei Hospital Taipei Taiwan; ^8^ Division of General Internal Medicine Zuckerberg San Francisco General Hospital University of California, San Francisco San Francisco, CA United States; ^9^ School of Public Health University of Sydney Sydney Australia

**Keywords:** health literacy, digital intervention, diabetes, quantitative trial, physical activity

## Abstract

**Background:**

Developing accessible Web-based materials to support diabetes self-management in people with lower levels of health literacy is a continuing challenge.

**Objective:**

The objective of this international study was to develop a Web-based intervention promoting physical activity among people with type 2 diabetes to determine whether audiovisual presentation and interactivity (quizzes, planners, tailoring) could help to overcome the digital divide by making digital interventions accessible and effective for people with all levels of health literacy. This study also aimed to determine whether these materials can improve health literacy outcomes for people with lower levels of health literacy and also be effective for people with higher levels of health literacy.

**Methods:**

To assess the impact of interactivity and audiovisual features on usage, engagement, and health literacy outcomes, we designed two versions of a Web-based intervention (one interactive and one plain-text version of the same content) to promote physical activity in people with type 2 diabetes. We randomly assigned participants from the United Kingdom, Austria, Germany, Ireland, and Taiwan to either an interactive or plain-text version of the intervention in English, German, or Mandarin. Intervention usage was objectively recorded by the intervention software. Self-report measures were taken at baseline and follow-up (immediately after participants viewed the intervention) and included measures of health literacy, engagement (website satisfaction and willingness to recommend the intervention to others), and health literacy outcomes (diabetes knowledge, enablement, attitude, perceived behavioral control, and intention to undertake physical activity).

**Results:**

In total, 1041 people took part in this study. Of the 1005 who completed health literacy information, 268 (26.67%) had intermediate or low levels of health literacy. The interactive intervention overall did not produce better outcomes than did the plain-text version. Participants in the plain-text intervention group looked at significantly more sections of the intervention (mean difference –0.47, 95% CI –0.64 to –0.30, P<.001), but this did not lead to better outcomes. Health literacy outcomes, including attitudes and intentions to engage in physical activity, significantly improved following the intervention for participants in both intervention groups. These improvements were similar across higher and lower health literacy levels and in all countries. Participants in the interactive intervention group had acquired more diabetes knowledge (mean difference 0.80, 95% CI 0.65-0.94, P<.001). Participants from both groups reported high levels of website satisfaction and would recommend the website to others.

**Conclusions:**

Following established practice for simple, clear design and presentation and using a person-based approach to intervention development, with in-depth iterative feedback from users, may be more important than interactivity and audiovisual presentations when developing accessible digital health interventions to improve health literacy outcomes.

**ClinicalTrial:**

International Standard Randomized Controlled Trial Number (ISRCTN): 43587048; http://www.isrctn.com/ISRCTN43587048. (Archived by WebCite at http://www.webcitation.org/6nGhaP9bv)

## Introduction

Health literacy has been defined as “knowledge, motivation and competences to access, understand, appraise, and apply health information” [1]. The capacity to understand and apply health information depends not only on the capabilities of the individual, but also on the way in which health information is presented. Well-designed materials to support self-management of health can help to improve health literacy outcomes such as knowledge, motivation, confidence, and adherence [2,3]. Lower levels of health literacy are associated with poor illness management, health knowledge, health service use, and health and with higher mortality. Addressing the challenges posed by low health literacy in populations has been highlighted as an urgent priority in many countries [4].

Barriers to accessing support for self-management of chronic health problems include disability, cost, work or family responsibilities, and lack of transport [5]. Studies have shown that these barriers are more common among people with lower levels of education [6]. Web-based health interventions may help address this problem, as they can be conveniently accessed in the home and reach large numbers of people at low cost, thereby having the potential to reduce health disparities [7]. Access to and use of the Internet through a personal computer or mobile phone is rapidly becoming common among more sections of the population, with over 80% of the adult population now using the Internet in the countries participating in this study [8]. However, low health literacy levels may present barriers to understanding and applying health information obtained from the Internet [9-11]. Lower levels of eHealth literacy are also associated with lower levels of healthy behavior, such as physical activity [12]. Therefore, reducing the “literacy burden” of online health information is an important strategy in making support for self-management of chronic conditions more accessible.

To date, interventions to reduce the literacy burden and improve health literacy have included using simple language, audiovisual or pictorial formats, interactivity, and tailoring of content to individuals’ needs (if the intervention is Web based). Reviews of the effectiveness of such interventions for the general public and mixed-patient populations [13-17] and for diabetes [18-20] suggest that these techniques show promise for some outcomes, but that overall the evidence for improving health literacy or reducing the literacy burden is weak and inconclusive, and it remains unclear exactly which elements of such interventions improve which outcomes.

This study addressed the evidence gap regarding how best to design Web-based materials for the growing population of patients with basic literacy and computer skills but lower levels of health literacy. We developed a Web-based intervention to promote physical activity in people with type 2 diabetes, following established best practice for designing accessible Web-based written content. We included a range of interactive elements (quiz, tailoring, a planner) and audiovisual modes of presentation, so that we could evaluate whether these improved usage and health literacy outcomes, particularly in those with lower levels of health literacy. We used our person-based approach to intervention development [21], carrying out iterative qualitative research with people with high and low levels of health literacy to gain feedback to improve accessibility and engagement [22].

This paper reports on a subsequent large international quantitative study comparing this Web-based intervention with a static, plain-text presentation of identical content. The study evaluated engagement and heath literacy outcomes in people with varied levels of health literacy. We measured engagement by objectively recorded intervention usage and self-reported user experience (website satisfaction and whether participants would recommend the website to others) [23]. The primary research question asked whether an interactive, tailored, and audiovisual Web-based intervention would lead to better engagement than a plain-text version of the same content. Secondary research questions asked (1) whether we could design a Web-based intervention that people with lower and higher levels of health literacy find engaging, (2) whether these materials could improve health literacy outcomes for people with lower levels of health literacy, and (3) whether the materials would also be effective for people with higher levels of health literacy.

## Methods

### Intervention Development

*Healthy Living with Diabetes* is a tailored Web-based intervention to motivate people with type 2 diabetes to increase their physical activity. The intervention was developed by a team of health researchers at the University of Southampton, United Kingdom, in collaboration with the Diabetes Literacy research consortium [24], patient representatives, and an international expert panel.

We developed 2 Web-based interventions using the LifeGuide software, an open access platform for developing Web-based behavior change interventions [25]. The first was a plain-text version of the intervention, and the second was an interactive version of the intervention. Both versions included the same content, which was written and designed to be accessible for people with lower levels of health literacy and to be engaging and novel. To enhance engagement, the intervention content contained novel and compelling information about the benefits of physical activity for people with type 2 diabetes. To enhance accessibility, we followed good practice guidelines for accessible Web-based design and presentation of written content [26-31] in both interventions.

We designed the interactive version to assess the additional impact that interactivity, audiovisual features, and tailoring may have on engagement with the intervention and health literacy outcomes in people with varied health literacy levels. Audiovisual aspects of the interactive intervention were positive images throughout, and a series of audiovisual sequences demonstrating lifestyle and physical activities (tailored to age and sex). The interactive features of the website consisted of a quiz, a physical activity planner, and tailored advice, feedback, and images based on user responses to questions (such as current physical activity levels, attitudes to physical activity, age, and sex).

We first developed the intervention in English for testing in the United Kingdom, and then adapted and translated it for testing in Austria, Germany, Ireland, Taiwan, and the United States. Researchers in the United States did not take part in this subsequent trial. We followed our person-based approach to intervention development [21,32] to enhance acceptability and feasibility from the earliest stages of intervention development through an in-depth understanding of the views and perspectives of our target users. Full details of the development and qualitative evaluation of the intervention, including screenshots of the intervention, have previously been published [22].

### Design

We carried out a multisite randomized trial in the United Kingdom, Austria, Germany, Ireland, and Taiwan to compare the interactive Web-based materials versus a plain-text Web-based version of the intervention. The plain-text intervention contained the same content and structure as the interactive version, but all tailoring, interactivity, and audiovisual features were removed. Ethics and research governance approvals were granted by the University of Southampton and UK National Health Service (NHS) research ethics committees (number 13/LO/0316).

### Participants and Procedure

Participants were invited to take part in the study if they were over 18 years old with a diagnosis of type 2 diabetes, had access to the Internet, were able to read the intervention language (English, German, or Mandarin), and give informed consent. We recruited participants from the United Kingdom, Austria, Germany, Ireland, and Taiwan between July 2014 and March 2015. Minor country differences in recruitment procedures were permitted to allow for differing health care systems and patient access. UK participants were recruited through 43 primary care practices specifically selected for being in areas of high deprivation in order to reach more people with low health literacy. Participants in Ireland and Taiwan were recruited opportunistically by health care professionals in diabetes outpatient clinics, and participants in Austria and Germany were recruited through national diabetes support group newsletters and advertisements placed on the Internet. Health care professionals in the United Kingdom, Ireland, and Taiwan screened potential participants to exclude patients with potential difficulties, including severe mental health problems, palliative care, recent bereavement, and inability to complete research measures (eg, learning disability, inability to read or speak an intervention language) before they were invited to the study.

Participants from all countries were presented with details of the study, research team contact details for more information, and a website URL where they could log in to the Web-based intervention on their own time. Participant information stated that we were comparing two types of webpages to see which was more helpful; it did not mention website features such as interactivity or audiovisual features. Participants were therefore blinded to what the differences between the 2 arms were. Consent was given online, and participants completed a very brief baseline questionnaire before being randomly assigned to 1 of the 2 groups (with a 50% ratio). Participants were then presented with either the interactive or plain-text Web-based materials, depending on randomization assignment. Participants were asked follow-up questions immediately after using the intervention. All recruitment and follow-up procedures (including full study information, obtaining informed consent, baseline and follow-up data collection, and randomization) were Web based using automated procedures carried out by the LifeGuide software [25].

### Sample Size

We calculated the sample size a priori using the G*Power 3 (version 3.1.9.2) sample size calculation program [33]. We calculated that a minimum sample size of 676 participants in total would be required to detect a small difference (effect size, Cohen *d*=0.25) between the 2 groups on our primary outcome measure of objective intervention usage, with alpha=.05 and beta=.1.

### Measures

Participants completed Web-based assessments at baseline (immediately before) and follow-up (immediately after using the intervention materials). We collected demographic variables at baseline, consisting of age, sex, time since diabetes diagnosis, and age they left full-time education. Participants’ levels of physical activity during the previous week were measured at follow-up using the International Physical Activity Questionnaire-Short Form (IPAQ-SF) self-administered questionnaire assessing the minutes spent doing vigorous and moderate activity and walking in the last 7 days [34]. We scored the IPAQ-SF using the recommended categorical scoring system [35], where participants are categorized as being either (1) inactive, (2) minimally active, or (3) highly active.

We measured engagement with the Web-based intervention by intervention usage and self-reported measures of engagement. Intervention usage was measured by the number of intervention sections completed, as total time spent on the intervention was likely to be confounded with format (plain text vs interactive). Both the interactive and plain-text intervention were designed to comprise 5 distinct sections: knowledge of physical activity benefits (with/without interactive quiz); advice on selecting physical activities (with/without tailoring); advice on planning physical activity (with/without interactive planner); success stories (with/without audiovisual presentation); access to further information about undertaking physical activity. All intervention usage data was automatically recorded by the LifeGuide software [25]. Self-reported measures of engagement at follow-up were a previously validated 3-item measure of satisfaction with Web-delivered advice [36], and a single item measuring whether participants would recommend the website to friends and family with diabetes, based on the NHS Friends and Family Test [37].

Health literacy outcomes were (1) diabetes knowledge, (2) patient enablement, and (3) attitude, behavioral control, and intention to undertake physical activity. Diabetes knowledge was measured by a 9-item knowledge quiz based on the intervention content. Patient enablement was measured by 3 items from the Patient Enablement Instrument [38] assessing participants’ perceptions of their understanding of the benefits of physical activity for people with diabetes, their ability to cope with diabetes, and confidence in managing their health. Participants completed these measures immediately after viewing the intervention. Attitude, behavioral control, and intention to undertake physical activity were measured by 3 items drawn from the theory of planned behavior [39]. Participants completed these items at baseline (immediately before viewing the intervention) and follow-up (immediately after completing the intervention) in order to assess change. Responses were given on a 7-point Likert scale (ranging from disagree to agree). These 3 items were (1) “Increasing my level of physical activity would be good for me” (physical activity attitude), (2) “I would find it easy to increase my level of physical activity” (perceived behavioral control), and (3) “I plan to increase my level of physical activity” (physical activity intentions).

We measured health literacy at baseline by a validated single item: “How often do you have problems learning about your condition because of difficulty understanding written information?” [40]. On the basis of this measure, we identified participants as having high, intermediate, or low levels of health literacy. Measures were translated from English to German and Mandarin and checked by each country’s research team for accuracy. All measures were optional apart from age and sex, which were essential for tailoring.

### Analysis

We analyzed the data using IBM SPSS for Windows version 14.0 (IBM Corporation) and Stata statistical software Special Edition Release 2007 (version 13; StataCorp LP), following a prespecified data analysis plan developed with our statistician (BS) and approved by the whole Diabetes Literacy consortium. All comparisons of the plain-text and interactive versions of the website controlled for potential confounding effects of the covariates health literacy, education, age, sex, and illness duration. We allowed for clustering by country by including country as a random effect in the model.

Due to the small numbers of participants with low health literacy levels, we categorized health literacy as low/intermediate compared with high health literacy. To avoid undertaking too many between-country comparisons, analyses by country compared UK data with a pooled sample of all other countries, as the UK sample was the largest and the intervention materials were originally developed for testing in the United Kingdom, and then translated and adapted for other countries and cultures.

The primary research question asked whether an interactive, tailored, and audiovisual Web-based intervention can lead to better engagement than a plain-text version of the same content can. The primary analysis compared the number of intervention sections completed by participants randomly assigned to the interactive intervention versus the number completed by participants randomly assigned to the plain-text intervention to test the prediction that more sections of the interactive version of the Web-based intervention would be completed. We used linear regression to compare the mean difference between intervention groups. We then examined whether intervention usage was moderated by health literacy level or by country. For these analyses, we carried out linear regressions to look for group differences by health literacy level and country. Post hoc exploratory analyses of Web usage were carried out using visualization analyses to examine patterns of intervention usage. Intervention usage data were analyzed using the LifeGuide visualization tool [41] to explore patterns of intervention use. This tool enables researchers to visualize and compare which intervention features were viewed, for how long, and in what order, across all participants.

Secondary research questions asked whether people with high and low health literacy found the materials engaging, and whether the intervention improved health literacy outcomes in people with lower and high levels of health literacy. In order to answer these questions, we analyzed self-report measures of engagement (website satisfaction; recommending the website to others) and health literacy outcomes (diabetes knowledge; patient enablement; and change in attitude, behavioral control, and intention to undertake physical activity) using linear regression models and then assessed for potential moderator effects by heath literacy level and country.

### Missing Data

The main outcome for this study was intervention usage, which was automatically recorded by the intervention software for all participants and therefore had no missing data. We investigated levels of missing data for baseline and follow-up measures and compared the frequency of missing data between the 2 intervention groups. Levels of missing data were high for the diabetes knowledge quiz score (459/1041, 44.09% missing) and the single item measuring whether participants would recommend the intervention to others (231/1041, 22.19% missing data). We assumed that these were at random and applied a multiple imputation model of 100 imputations for missing secondary outcomes and key covariates. We present this analysis as a sensitivity analysis alongside the main analysis on complete cases.

## Results

### Participants

**Figure 1:**
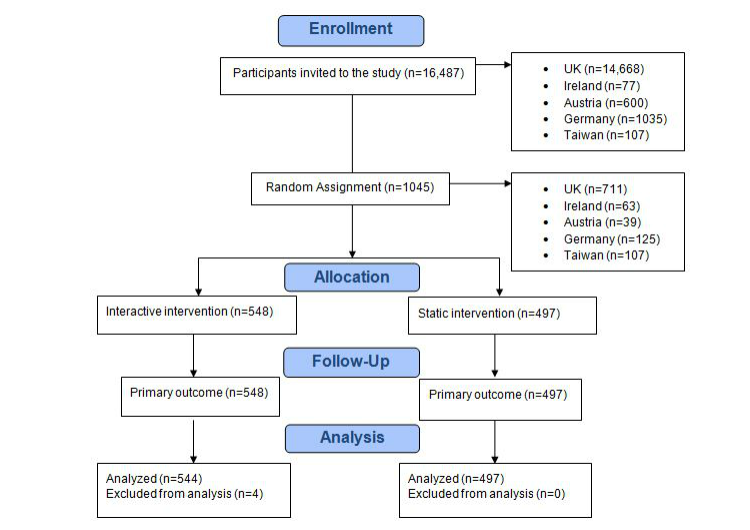
Consolidated Standards of Reporting Trials (CONSORT) flow diagram.

In total, 1045 participants from the United Kingdom, Austria, Germany, Ireland, and Taiwan participated in the study and were randomly assigned to view either the interactive intervention or the plain-text intervention. Of these, 4 participants used the Back button on their Internet browsers to be rerandomized and were consequently excluded, resulting in 1041 participants in the final analysis. We successfully measured the primary outcome, intervention usage, for 100% of randomly assigned participants. See Figure 1 for the Consolidated Standards of Reporting Trials (CONSORT) flow diagram.

### Participant Characteristics

Participants in this study were predominantly male (662/1041, 63.59%), with a mean age of 62 years. On average, participants left full-time education before the age of 18 years and had been diagnosed with type 2 diabetes for 9.2 years (this ranged from just a few months to 50 years). The majority of participants (737/1005, 73.33%) had high levels of health literacy, while 268/1005 (26.67%) had intermediate or low levels of health literacy. A total of 835/1041 (80.21%) of participants completed the IPAQ-SF physical activity questionnaire. Most of these participants reported being inactive (561/835, 67.2%), while some reported being minimally active (190/835, 22.8%) and a minority reported being highly active (84/835, 10.1%). Participant characteristics were similar across both groups at baseline, with the only slight difference being higher health literacy levels in the interactive group. See Table 1 for participant characteristics by intervention group. Participant characteristics were similar by country (see Multimedia Appendix 1 for details).

**Table 1:** Participant characteristics in the 2 arms of the Web-based intervention promoting physical activity among people with type 2 diabetes.

Characteristic		Group	
		Plain-text (n=497)	Interactive (n=544)
Female, n (%)		182 (36.6)	197 (36.2)
Age in years, mean (SD)		61.5 (11.2)	62.4 (11.4)
Years since diagnosis, mean (SD)		9.1 (9.1)	9.5 (9.3)
Age when left full-time education, mean (SD)		17.8 (3.0)	17.8 (3.0)
**Health literacy level (single-item measure), n (%)**			
	Low	37/478 (7.7)	30/527 (5.7)
	Intermediate	105/478 (22.0)	96/527 (18.2)
	High	336/478 (70.3)	401/527 (76.1)
**Physical activity attitudes and intentions**			
	IPAQ-SF^a^, mean (SD)	15.1 (3.5)	15.0 (3.7)
	Highly active, n (%)	35/431 (8.1)	49/404 (12.1)
	Minimally active, n (%)	106/431 (24.6)	84/404 (20.8)
	Inactive, n (%)	290/431 (67.3)	271/404 (67.1)

^a^IPAQ-SF: International Physical Activity Questionnaire-Short Form.

### Intervention Usage

The primary outcome in this study was Web-based intervention usage to test whether the interactive intervention led to better engagement than the plain-text version. Analysis of usage data found a significant difference in intervention usage between the 2 groups, with participants in the interactive intervention group being likely to complete fewer of the 5 intervention sections than were participants in the plain-text intervention group (mean difference –0.47, 95% CI –0.64 to –0.30, *P*<.001). Table 2 gives the results of intervention usage analyses.

Moderator analysis examined intervention usage by health literacy level. Participants with higher levels of health literacy were significantly more likely to complete more sections of the intervention (mean difference 0.25, 95% CI 0.05-0.45, *P*=.02; Table 3).

**Table 2:** Results of analysis of intervention usage as determined by number of sections completed, and results of self-reported measures of engagement and moderator analyses of self-reported engagement, by intervention group.

Analysis		Intervention group		Univariate difference		Multivariate difference^a^		Multivariate difference^a^ based on 100 imputations	
		Plain text	Interactive	Mean (95% CI)	*P* value	Mean (95% CI)	*P* value	Mean (95% CI)	*P* value
**Intervention usage**									
	No. of sections completed, mean (SD)	4.5 (1.3)	4.0 (1.5)	–0.47 (–0.64 to –0.30)	<.001	–0.49 (–0.67 to –0.31)	<.001	N/A^b^	N/A
**Measures of engagement**									
	Satisfied with website, mean (SD)	4.1 (2.0)	4.1 (1.9)	0.03 (–0.24 to 0.30)	.82	0.05 (–0.22 to 0.33)	.70	0.08 (–0.19 to 0.35)	.54
	Would recommend to others, n (%)	281/419 (67.1)	248/391 (63.4)	0.85 (0.64 to 1.14)	.28	0.85 (0.62 to 1.15)	.29	0.78 (0.58 to 1.05)	.10

^a^All analyses controlled for possible confounding by age, sex, time since diagnosis, age when the participant left education, health literacy, and for clustering by country.

^b^N/A: not applicable.

**Table 3:** Results of analysis of intervention usage as determined by number of sections completed, and results of self-reported measures of engagement and moderator analyses of self-reported engagement, by health literacy level.

Analysis		Health literacy level		Univariate difference		Multivariate difference^a^		Multivariate difference^a^ based on 100 imputations	
		Lower	High	Mean (95% CI)	*P* value	Mean (95% CI)	*P* value	Mean (95% CI)	*P* value
**Intervention usage**									
	No. of sections completed, mean (SD)	4.1 (1.5)	4.3 (1.4)	0.25 (0.05 to 0.45)	.02	0.28 (0.08 to 0.48)	.01	N/A^b^	N/A
**Measures of engagement**									
	Satisfied with website, mean (SD)	4.1 (2.0)	4.1 (2.0)	–0.03 (–0.34 to 0.29)	.87	0.05 (–0.27 to 0.37)	.76	0.04 (–0.28 to 0.35)	.82
	Would recommend to others, n (%)	139/195 (71.3)	372/591 (62.9)	0.70 (0.48 to 0.97)	.04	0.64 (0.44 to 0.93)	.02	0.69 (0.48 to 1.01)	.05

^a^All analyses controlled for possible confounding by age, sex, time since diagnosis, age when the participant left education, health literacy, and for clustering by country.

^b^N/A: not applicable.

**Figure 2:**
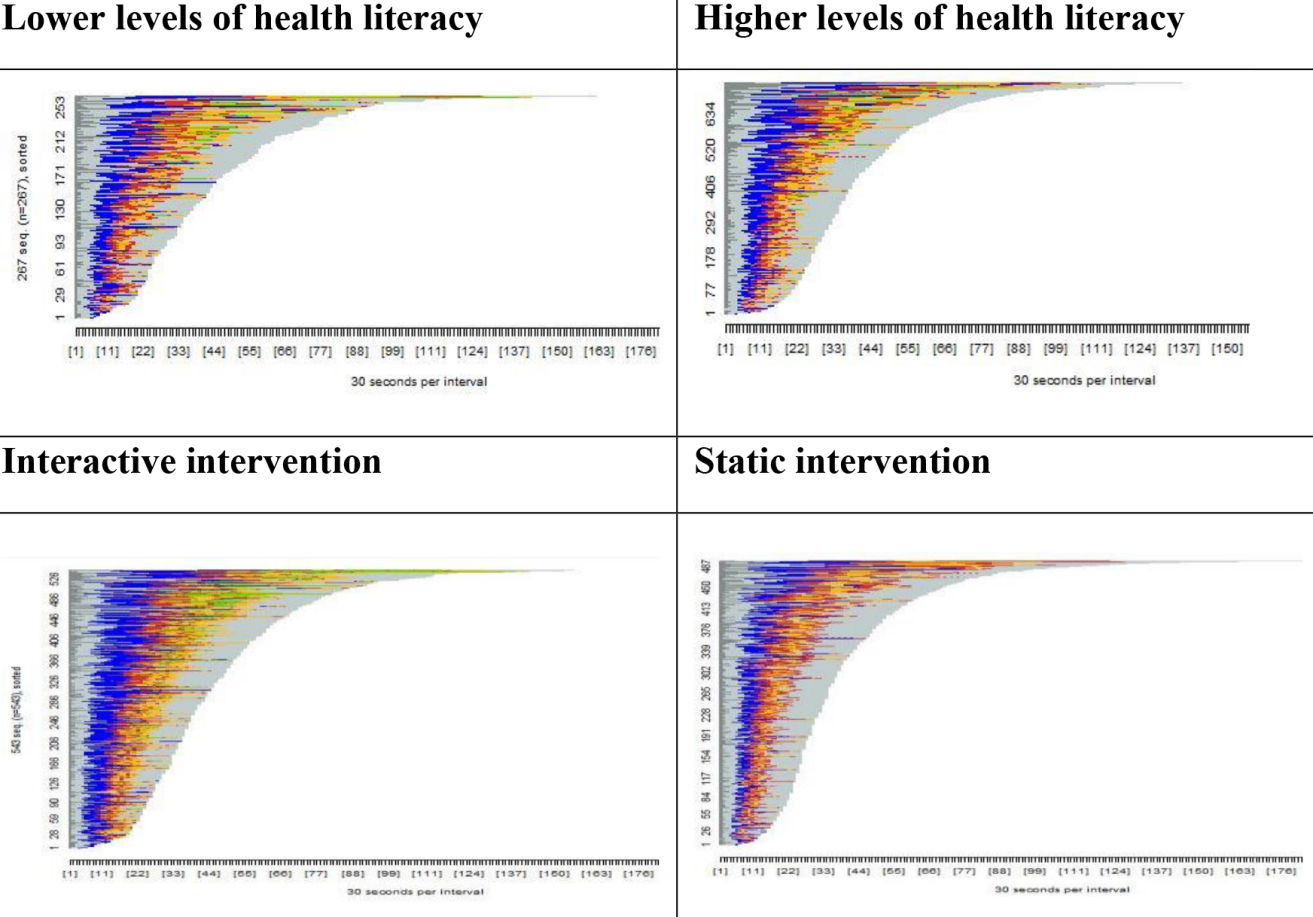
Visualization of intervention usage by health literacy level and intervention. Blue: time spent on quiz; yellow: time spent on physical activity planner; red: time spent on reading personal tips; green: time spent on audiovisual sequences.

We carried out exploratory analyses to examine whether intervention usage differed by country (comparing the United Kingdom versus the other participating countries). Patterns of usage were similar in the United Kingdom and the other countries. See Multimedia Appendix 2 for details of intervention usage by country. Extensive visualization analyses of usage in the whole sample revealed no difference in how the intervention was used by health literacy level, age, sex, time since diagnosis, physical activity level, or change in physical activity attitude. Visualization analyses outputs illustrate intervention usage for the selected sample over time (in 30-second intervals). Visualization analyses comparing usage of the interactive intervention and the static intervention revealed that the interactive group spent more time on the quiz (represented by blue in Figure 2) and the physical activity planner (represented by yellow), while the static intervention group spent more time reading personal tips (represented by red). These differences could be explained by the interactive nature of the quiz and planner adding to the time taken to complete them, while the lack of tailoring in the static intervention increased the reading burden for the personal tips section (since all tips were presented rather than just those tailored to the users). Similarly, we did not include audiovisual sequences (represented by green) in the static intervention and these are therefore represented only in the visualization of usage of the interactive intervention. See Figure 2 for visualization of intervention usage by health literacy level and intervention.

### Self-Reported Measures of Engagement

The self-reported measures of engagement were website satisfaction and a single item measuring whether participants would recommend the website to others. We used these items to address the secondary research question asking whether Web-based materials can be developed to be engaging to people with low and high levels of health literacy. There were no significant group differences, with participants in both groups reporting high levels of website satisfaction and the majority of participants in both groups reporting that they would be likely to recommend the website to others. Table 2 and Table 3 give details of these results.

We carried out exploratory analyses to evaluate whether self-reported measures of engagement varied by health literacy level. Participants with lower health literacy were significantly more likely to recommend the website to friends or family with diabetes (mean difference –0.70, 95% CI 0.48-0.97, *P*=.04), although this difference was no longer significant following 100 imputations (mean difference 0.69, 95% CI 0.48-1.01, *P*=.05). There were no significant differences in website satisfaction, with participants with all levels of health literacy reporting high levels of satisfaction. See Table 3 for details. Moderator analysis found that the same pattern of results occurred in the UK data compared with other countries. See Multimedia Appendix 3 for details of moderator analyses by country.

### Health Literacy Outcomes

Secondary research questions asked whether the Web-based materials could improve health literacy outcomes in people with low health literacy and be effective for people with higher levels of health literacy. The health literacy outcomes in this study were (1) diabetes knowledge, (2) patient enablement, and (3) change in attitude, behavioral control, and intention to undertake physical activity. There was a significant group difference in participants’ diabetes knowledge, with participants in the interactive group scoring significantly higher than the plain-text intervention group (mean difference 0.80, 95% CI 0.65-0.94, *P*<.001). The diabetes knowledge measure had a ceiling effect with a large proportion of participants from both groups scoring highly. When comparing participants who answered all the questions correctly with those who got 1 or more wrong, the group difference was maintained and the interactive group was nearly 7 times more likely than the plain-text group to have answered all the questions correctly (mean difference 6.5, *P*<.001, 95% CI 4.4-9.4). There were no significant group differences in patient enablement, with participants in both groups reporting feeling more enabled as a result of using the intervention materials. Details of these results are given in Table 4.

**Table 4:** Health literacy outcomes by intervention group.

Outcome	Intervention group		Univariate difference		Multivariate difference^a^		Multivariate difference^a^ based on 100 imputations	
	Plain text	Interactive	Mean (95% CI)	*P* value	Mean (95% CI)	*P* value	Mean (95% CI)	*P* value
Diabetes knowledge, mean (SD)	8.0 (1.1)	8.8 (0.5)	0.80 (0.65 to 0.94)	<.001	0.78 (0.63 to 0.92)	<.001	0.74 (0.50 to 0.88)	<.001
Diabetes knowledge score of 9 vs lower score, n (%)	124/303 (40.9)	228/279 (81.7)	6.5 (4.4 to 9.4)	<.001	6.9 (4.6 to 10.3)	<.001	4.90 (3.35 to 7.17)	<.001
Patient Enablement Instrument, mean (SD)	7.5 (3.1)	7.6 (3.0)	0.08 (–0.33 to 0.49)	.70	0.02 (–0.40 to 0.43)	.93	0.17 (–0.25 to 0.58)	.44

^a^All analyses controlled for possible confounding by age, sex, time since diagnosis, age when the participant left education, health literacy, and for clustering by country.

Moderator analyses explored these results by health literacy level. There was a trend for people with higher levels of health literacy to score higher on the Patient Enablement Instrument (multivariate mean difference 0.53, 95% CI 0.04-1.02, *P*<.03), although this was no longer significant following 100 imputations (mean difference 0.40, 95% CI –0.09 to 0.88, *P*<.11). There were no significant health literacy differences in diabetes knowledge acquired, with both groups scoring highly. See Table 5 for details. Moderation analyses by country showed a similar pattern of results for the United Kingdom compared with other countries; see Multimedia Appendix 4 for details.

Participants were asked about their attitudes and intentions toward physical activity at baseline and again at follow-up, enabling an analysis to establish whether the score had changed within each group. In both intervention groups, and across all health literacy levels, the score at follow-up was significantly higher than at baseline, indicating that participants from all groups had more positive attitudes and intentions toward physical activity after viewing the intervention materials. Table 6 shows the results of this analysis.

**Table 5:** Moderator analyses of health literacy outcomes by health literacy levels.

Outcome	Health literacy level		Univariate difference		Multivariate difference^a^		Multivariate difference^a^ based on 100 imputations	
	Lower	High	Mean (95% CI)	*P* value	Mean (95% CI)	*P* value	Mean (95% CI)	*P* value
Diabetes knowledge, mean (SD)	8.2 (1.1)	8.4 (0.9)	0.16 (–0.02 to 0.35)	.09	0.13 (–0.05 to 0.30)	.16	0.13 (–0.06 to 0.32)	.18
Diabetes knowledge score of 9 vs lower score, n (%)	73/132 (55.3)	270/434 (62.2)	1.33 (0.90 to 1.97)	.16	1.31 (0.83 to 2.07)	.25	1.27 (0.84 to 1.92)	.26
Patient Enablement Instrument, mean (SD)	7.3 (2.8)	7.7 (3.1)	0.39 (–0.09 to 0.87)	.11	0.53 (0.04 to 1.02)	.03	0.40 (–0.09 to 0.88)	.11

^a^All analyses controlled for possible confounding by age, sex, time since diagnosis, age when the participant left education, and for clustering by country.

**Table 6:** Change in attitude behavioral control and physical activity intentions from baseline to follow-up across all groups and literacy levels.

Outcome	Plain text group		Interactive group		Lower health literacy		High health literacy	
	Mean (95% CI)	*P* value	Mean (95% CI)	*P* value	Mean (95% CI)	*P* value	Mean (95% CI)	*P* value
Physical activity attitude	0.10 (0.02-0.18)	.01	0.22 (0.11-0.34)	<.001	0.15 (0.02-0.27)	.02	0.15 (0.07-0.23)	<.001
Perceived behavioral control	0.34 (0.24-0.45)	.001	0.35 (0.22-0.47)	<.001	0.33 (0.17-0.49)	<.001	0.34 (0.24-0.43)	<.001
Physical activity intention	0.35 (0.24-0.45)	<.001	0.49 (0.35-0.63)	<.001	0.27 (0.10-0.44)	.002	0.46 (0.35-0.56)	<.001

## Discussion

### Principal Findings

The main finding of this study was that the interactive intervention overall did not produce better outcomes than those obtained by a plain-text version of the intervention. Participants in the plain-text intervention group showed higher levels of engagement by completing more sections of the intervention, although this did not lead to better health literacy outcomes, and participants in the interactive intervention group had better diabetes knowledge.

Health literacy outcomes significantly improved following the intervention to a very similar extent in both groups. These significant changes were reflected across all health literacy levels and all countries, with participants reporting increased beliefs in the benefits of physical activity, greater confidence in undertaking physical activity, and a stronger intention to increase physical activity as a result of the intervention. Given the low levels of physical activity reported by our sample, these changes in attitude to physical activity are positive, and it is encouraging that we observed these changes in those with lower levels of health literacy, since low self-confidence for physical activity has been shown to be a key mediator of the association between low health literacy and inactivity [42]. Diabetes knowledge was higher in the interactive group, suggesting that the interactive quiz format may have been useful for learning new information. Both interactive and plain-text intervention groups reported high levels of enablement as a result of viewing the intervention materials, and both intervention groups were likely to recommend the intervention to friends or family with diabetes.

Analysis by health literacy level revealed few differences. Participants with high levels of health literacy completed more sections of the intervention, but this did not lead to better health literacy outcomes. Participants with high health literacy reported higher levels of enablement, and participants with lower health literacy were more likely to recommend the intervention to others, but these differences were not significant after correcting for missing data. Despite these minor group differences, there are encouraging signs that the intervention design was accessible and helpful for people with all health literacy levels. These findings are consistent with evidence from previous research that interventions designed to be accessible for people with lower health literacy can be suitable for people with higher health literacy [15,22,43]. Participants with all health literacy levels reported high levels of enablement and were likely to recommend the intervention to friends or family members with diabetes. We observed similar patterns of results in the United Kingdom compared with other countries, suggesting the translated and adapted materials were equally effective. A detailed description and illustrations of the intervention have previously been published [22].

However, more work is needed to engage hard-to-reach populations in Web-based interventions. Despite deliberately sampling in socially deprived populations, we attracted surprisingly few people with lower levels of health literacy.

### Limitations

This study did not succeed in recruiting many participants with very low levels of health literacy, and the results can therefore not be generalized to this group. It is also important to note that the results only refer to our version of interactivity, and others may be able to produce more engaging interactive materials. This study was not powered for examining interactions, and all subgroup analyses were exploratory and should be interpreted with caution. There were minor recruitment differences between countries, which should be taken into account when interpreting response rates. We did not undertake longer-term follow-up and therefore do not know the extent to which the immediate intervention effect will endure in this population. Since this study did not include a control group, we cannot draw firm conclusions regarding the effectiveness of the Web-based intervention content, since changes in attitudes before and after viewing the content could in theory have been due to other factors.

### Conclusion

In this study, a good, clear design and person-based intervention development [21,32] to establish an in-depth understanding of the views and perspectives of target users appears to have been more important than interactivity and audiovisual presentation when developing accessible digital health interventions to improve health literacy outcomes. This approach also seems able to be adapted for successful use in different counties and cultures. The finding that the same materials can be equally engaging for people with high and lower levels of health literacy is important, since the need to tailor or target interventions for different sectors of the population increases the complexity of interventions and could reduce their cost effectiveness. Consequently, well-designed digital communication materials that have been developed and evaluated for accessibility with a range of users may be sufficient as a means of filling unmet needs for improving health literacy. Looking to the future, more needs to be done to encourage and support intervention providers to develop Web-based materials that can benefit people with limited health literacy.

## Appendix

Multimedia Appendix 1 Participant characteristics by country.

Multimedia Appendix 2 Moderator analysis of intervention usage by country.

Multimedia Appendix 3 Moderator analyses of self-reported engagement by country.

Multimedia Appendix 4 Moderator analyses of health literacy outcomes by country.

Multimedia Appendix 5 CONSORT-EHEALTH checklist V1.6.2 [44].

## Abbreviations

CONSORT: Consolidated Standards of Reporting Trials
IPAQ-SF: International Physical Activity Questionnaire-Short Form
NHS: National Health Service
