# Supplementary material for: Effects on Engagement and Health Literacy Outcomes of Web-Based Materials Promoting Physical Activity in People With Diabetes: An International Randomized Trial
Source: J Med Internet Res. 2017 Jan 23;19(1):e21. doi: 10.2196/jmir.6601 (PMC5294369; doi:10.2196/jmir.6601)
Supplement: Multimedia Appendix 1 [file jmir_v19i1e21_app1.pdf]

Multimedia Appendix 1. Participant characteristics by country

	UK	Ireland	Taiwan	Germany	Austria
Female	249/710 (35.1%)	20/61 (32.8%)	57/107 (53.3%)	40/125 (32%)	13/38 (34.2)
Age (mean (SD))	62.8 (10.2)	63.5 (10.5)	52.7 (15.3)	63.5 (10.1)	63.9 (12.3)
Years since diagnosis (mean (SD))	8.3 (7.2)	9.9 (6.3)	8.2 (7.7)	19.6 (14.3)	15.4 (12.8)
Age left full time education (mean (SD))	17.3 (2.7)	19.7 (3.2)	18.7 (4.1)	18 (2.8)	20.7 (3)
Health Literacy (single item measure)					
• Low	45/689 (6.5%)	8/56 (3.6%)	5/106 (4.7%)	12/119 (10.1%)	3/35 (8.6%)
• Intermediate	131/689 (19%)	8/56 (14.3%)	16/106 (15.1%)	34/119 (28.6%)	12/35 (34.3%)
• High	513/689 (74.5%)	46/56 (82.1%)	85/106 (80.2%)	73/119 (61.3%)	20/35 (57.1)
Physical Activity Attitudes and Intentions	15.0 (3.7)	16.0 (3.4)	15.9 (3.3)	14.4 (3.2)	13.9 (2.9)
IPAQ (mean (SD))					
• highly active	58/588 (9.9%)	3/53 (5.7%)	6/90 (6.7%)	14/83 (16.9%)	3/21 (14.3%)
• minimally active	126/588 (21.4%)	7/53 (13.2%)	31/90 (34.4%)	22/83 (26.5%)	4/21 (19%)
• inactive	404/588 (68.7%)	43/53 (81.1%)	53/90 (58.9%)	87/83 (56.6)	14/21 (66.7%)
