# Supplementary material for: Effects on Engagement and Health Literacy Outcomes of Web-Based Materials Promoting Physical Activity in People With Diabetes: An International Randomized Trial
Source: J Med Internet Res. 2017 Jan 23;19(1):e21. doi: 10.2196/jmir.6601 (PMC5294369; doi:10.2196/jmir.6601)
Supplement: Multimedia Appendix 2 [file jmir_v19i1e21_app2.pdf]

Multimedia Appendix 2. Moderator analysis of intervention usage by country

	UK only				Other countries			
	Plain text	Interactive	Univariate difference (95% CI)	Multivariate difference (95% CI)*	Plain text	Interactive	Univariate difference (95% CI)	Multivariate difference (95% CI)*
Usage: number of sections completed (mean (SD))	4.5 (1.3)	4.1 (1.4)	-0.45 (-0.66, -0.26; p<0.001)	-0.47 (-0.67, -0.26; p<0.001)	4.5 (1.4)	4.0 (1.6)	-0.50 (-0.84, -0.17; p=0.003)	-0.53 (-0.90, -0.17; p=0.004)
