# Supplementary material for: Effects on Engagement and Health Literacy Outcomes of Web-Based Materials Promoting Physical Activity in People With Diabetes: An International Randomized Trial
Source: J Med Internet Res. 2017 Jan 23;19(1):e21. doi: 10.2196/jmir.6601 (PMC5294369; doi:10.2196/jmir.6601)
Supplement: Multimedia Appendix 3 [file jmir_v19i1e21_app3.pdf]

Multimedia Appendix 3. Moderator analyses of self-reported engagement by country

	UK only				Other countries			
	Plain text	Interactive	Univariate difference (95% CI)	Multivariate difference (95% CI)*	Plain text	Interactive	Univariate difference (95% CI)	Multivariate difference (95% CI)*
Website satisfaction (mean (SD))	4.2 (2.1)	4.2 (1.9)	0.04 (-0.29, 0.37; p=0.799)	0.07 (-0.27, 0.40; p=0.685)	3.9 (1.8)	3.9 (1.7)	-0.04 (-0.49, 0.41; p=0.869)	-0.02 (-0.49, 0.44; p=0.918)
Would recommend to others	179/285 (63%)	170/283 (60%)	0.89 (0.64, 1.25; p=0.503)	0.90 (0.63, 1.26; p=0.538)	102/134 (76%)	78/108 (72%)	0.82 (0.46, 1.46; p=0.490)	0.70 (0.37, 1.31; p=0.262)

*All analyses control for possible confounding by age, gender, time since diagnosis, age left education, and for clustering by country.
