# Supplementary material for: Effects on Engagement and Health Literacy Outcomes of Web-Based Materials Promoting Physical Activity in People With Diabetes: An International Randomized Trial
Source: J Med Internet Res. 2017 Jan 23;19(1):e21. doi: 10.2196/jmir.6601 (PMC5294369; doi:10.2196/jmir.6601)
Supplement: Multimedia Appendix 4 [file jmir_v19i1e21_app4.pdf]

Multimedia Appendix 4. Moderator analyses of health literacy outcomes by country

	UK only				Other countries			
	Plain text	Interactive	Univariate difference (95% CI)	Multivariate difference (95% CI)*	Plain text	Interactive	Univariate difference (95% CI)	Multivariate difference (95% CI)*
Diabetes knowledge (mean (SD))	8.02 (1.2)	8.8 (0.4)	0.83 (0.65, 1.00; p<0.001)	0.83 (0.66, 1.00; p<0.001)	7.9 (1.0)	8.6 (0.8)	0.69 (0.42, 0.95; p<0.001)	0.68 (0.41, 0.95; p<0.001)
Diabetes knowledge score of 9 compared to lower score	89/202 (44%)	174/202 (86%)	7.89 (4.85, 12.83; p<0.001)	8.64 (5.17, 14.43; p<0.001)	35/101 (35%)	54/77 (70%)	4.43 (2.34, 8.37; p<0.001)	4.68 (2.36, 9.30; p<0.001)
Patient Enablement Instrument (mean (SD))	8.0 (3.2)	7.9 (3.1)	-0.12 (-0.63, 0.38; p=0.631)	-0.11 (-0.62, -0.40; p=0.664)	6.6 (2.7)	6.9 (2.6)	0.38 (-0.29, 1.06; p=0.264)	0.38 (-0.31, 1.07; p=0.285)

*All analyses control for possible confounding by age, gender, time since diagnosis, age left education, health literacy and for clustering by country.
